# The Role of Post-Mastectomy Radiotherapy in T1-2N1 Breast Cancer Patients: Propensity Score Matched Analysis

**DOI:** 10.3390/cancers15225473

**Published:** 2023-11-19

**Authors:** Kangpyo Kim, Won Park, Haeyoung Kim, Won Kyung Cho, Nalee Kim, Seok Jin Nam, Seok Won Kim, Jeong Eon Lee, Jonghan Yu, Byung Joo Chae, Se Kyung Lee, Jai Min Ryu

**Affiliations:** 1 Department of Radiation Oncology, Samsung Medical Center, Sungkyunkwan University School of Medicine, Seoul 06351, Republic of Korea; 2 Division of Breast Surgery, Department of Surgery, Samsung Medical Center, Sungkyunkwan University School of Medicine, Seoul 06351, Republic of Korea

**Keywords:** breast cancer, mastectomy, post-mastectomy radiotherapy, T1-2N1

## Abstract

**Simple Summary:**

With the development of modern systemic therapy, the role and indications of PMRT need to be further investigated in early-stage N1 breast cancer patients who have received upfront mastectomy, as recent guidelines still do not reach a consensus. Our study aimed to identify risk factors that may worsen treatment outcomes, and found that three lymph node metastases were prognostic for loco-regional control (LRC), disease-free survival (DFS), and lympho-vascular invasion (LVI) for overall survival (OS). However, the benefit of PMRT was not evident even in patients with these risk factors, and the results were similar after propensity score matching. Moreover, the incidence of arm lymphedema was significantly higher after PMRT. Therefore, we cautiously suggest omitting PMRT in T1-2N1 breast cancer patients, given the similar oncologic outcomes and increased risk of RT-related toxicity after PMRT.

**Abstract:**

This study aimed to evaluate the role of post-mastectomy radiotherapy (PMRT) in T1-2N1 breast cancer. Between 2006 and 2014, a total of 504 patients with T1-2N1 breast cancer were analyzed. PMRT was administered to 71 patients, and 1:2 propensity score matching (PSM) was performed between the PMRT and non-PMRT groups. Loco-regional control (LRC), disease-free survival (DFS), and overall survival (OS) rates were compared according to PMRT status. Thirteen and one loco-regional recurrences were observed in the PMRT and non-PMRT groups, respectively. Before PSM, the 8-year LRC, DFS, and OS rates in the non-PMRT and PMRT groups were 98.5% and 96.5% (*p* = 0.426), 89.7% and 91.2% (*p* = 0.700), and 91.5% and 92.1% (*p* = 0.679), respectively. Corresponding rates were 95.6% and 96.5% (*p* = 0.365), 84.1% and 91.2% (*p* = 0.185), and 88.4% and 92.1% (*p* = 0.276), respectively, after PSM. Multivariate analysis showed that three lymph node metastases were prognostic for LRC and DFS rates and LVI for OS rate. Arm lymphedema developed in 32.4% of patients who received PMRT, which was significantly higher than the non-PMRT group (*p* < 0.001). Contributions of PMRT for improvement of treatments outcomes in T1-2N1 breast cancer patients were not evident, while the incidence of arm lymphedema significantly increased after PMRT. Further prospective trials are required to re-evaluate the role of PMRT.

## 1. Introduction

Post-mastectomy radiotherapy (PMRT) has been administered to early breast cancer patients to reduce loco-regional recurrence (LRR) and cancer-specific mortality, supported by two landmark studies: The European Organization for Research and Treatment (EORTC) 22922/10925 trial [1] and the Early Breast Cancer Trialists’ Collaborative Group (EBCTCG) meta-analysis [2]. The National Comprehensive Cancer Network guidelines, therefore, strongly recommend chest wall and comprehensive elective nodal irradiation currently for patients with breast cancer with 1–3 lymph node (LN) metastases after mastectomy [3]. However, loco-regional control (LRC) and disease-free survival (DFS) rates have been improved along with the advances of systemic therapies, which are applied according to individual molecular profiles [4,5,6,7], and consequently, recent American and European consensus guidelines cautiously recommend omitting PMRT in patients with favorable disease characteristics [8,9], although the oncologic safety of this treatment de-escalation has not been confirmed.

A recent white paper of the Assisi Think Tank Meeting [10] stated that the committee did not reach agreement on PMRT in T2N1 patients after ALND. Furthermore, the guidelines do not indicate mandatory chest wall RT in patients who have not undergone ALND unless risk factors for relapse are present. In other words, the consensus of the meeting implies that more confirmative evidence is required to successfully superselect the indications of PMRT in N1 patients. To find out the appropriate indications of PMRT in T1-2N1 breast cancer patients, several randomized trials, including the MA20 and EORTC 22922/10925 trials, have established a “high-risk” category of regional failure that may benefit from regional nodal irradiation (RNI) [1,11], but the results are inconsistent [12,13]. The ongoing TAILOR-RT-NICI MA.39 trial (NCT03488693) will suggest an important clue and guideline for the indication of PMRT; however, this study is only focusing on patients with luminal type and low 21-gene Recurrence Score. This background emphasizes the needs of further investigation regarding the role of PMRT in early-stage N1 breast cancer patients.

We hypothesized that PMRT may possibly be omitted after total mastectomy in selected patients with T1-2N1 breast cancer treated with standard systemic therapies without compromising oncological outcomes. Therefore, we investigated the treatment outcomes and RT-related toxicities in patients who underwent upfront mastectomy with or without RT with modern systemic therapy regimens.

## 2. Materials and Methods

### 2.1. Patients

The inclusion criteria were pT1-2N1 invasive breast cancer patients who received upfront total mastectomy at Samsung Medical Center between 2006 and 2014. The exclusion criteria were as follows; ≥pN2 stage (*n* = 1263), immediate reconstruction (*n* = 330), T3-4 disease (*n* = 47), bilateral breast cancer (*n* = 44), immediate follow-up loss after surgical resection or not complete medical record (*n* = 36), and male breast cancer (*n* = 8). Finally, among 2232 patients who met the inclusion criteria, 504 patients were analyzed in the present study after exclusion. This study was approved by the Institutional Review Board of Samsung Medical Center (approval number 2023-01-100).

### 2.2. Treatment

Complete axillary LN dissection (ALND) is usually performed with total mastectomy. However, since 2009, adjuvant radiotherapy (RT) or close observation without further ALND has been recommended for patients with solitary micrometastasis on sentinel LN biopsy. Since 2011, PMRT involving the supraclavicular fossa has been indicated for patients presenting with two or more of the following risk factors based on institutional policy: >2 axillary LN metastases, axillary level II–III LN metastases, lympho-vascular invasion (LVI), or perinodal extension [14]. Internal mammary node (IMN) irradiation is not routinely performed. Three-dimensional conformal RT planning is performed with a prescribed dose of 50.0–50.4 Gy in 25–28 fractions.

Adjuvant chemotherapy with doxorubicin and cyclophosphamide (AC) followed by four cycles of docetaxel (T) is administered, except in patients with poor tolerance to adjuvant chemotherapy. Hormone or anti-HER2 therapy is also administered according to hormone receptor positivity and HER2 status.

### 2.3. Statistical Analysis

Patient characteristics were compared according to the use of PMRT using the chi-square test for categorical variables and the Mann–Whitney U test for continuous variables. Propensity score matching (PSM) was performed to compensate for selection bias and potential confounders between the treatment groups. Patients were matched in a 2:1 ratio using the nearest matching method within a caliper distance of 0.05. Propensity scores were calculated using a multivariate logistic regression model based on the following variables: age, tumor location, multifocality, tumor grade, LVI, molecular subtype, axillary management, and number of LN metastases.

The study endpoints were LRC, DFS, and overall survival (OS) rates. LRR was defined as local recurrence in the skin/chest wall or regional recurrence in the ipsilateral axillary, supraclavicular, infraclavicular, or IMNs. All endpoints were calculated from the date of surgery using the Kaplan–Meier method and compared using the log-rank test. Univariate and multivariate analyses of LRC, DFS, and OS rates were performed using the Cox proportional hazards model to identify risk factors for each outcome of interest. Statistical significance was defined as a two-sided *p* < 0.05. All statistical analyses were performed using SPSS version 27.0 (IBM Corp., Armonk, NY, USA) and R version 4.1.3 (Vienna, Austria; http://www.R-project.org/).

## 3. Results

### 3.1. Baseline Characteristics

The patient and tumor characteristics of the study population before and after PSM are summarized in Table 1. Compared to the non-PMRT group, patients in the PMRT group were more likely to have multifocal tumors (47.9% vs. 35.3%, respectively; *p* = 0.042), LVI (42.3% vs. 21.1%, respectively; *p* = 0.001), three LN metastases (42.3% vs. 11.5%, respectively; *p* < 0.001), and T2-stage disease (74.6% vs. 58.2%, respectively; *p* = 0.009). The likelihood of receiving taxane-based chemotherapy or endocrine therapy was also higher in the PMRT group. After PSM, there were 71 patients in the PMRT group and 142 patients in the non-PMRT group; the characteristics were well-balanced between the two groups.

### 3.2. Treatment Outcomes

**Figure 1:**
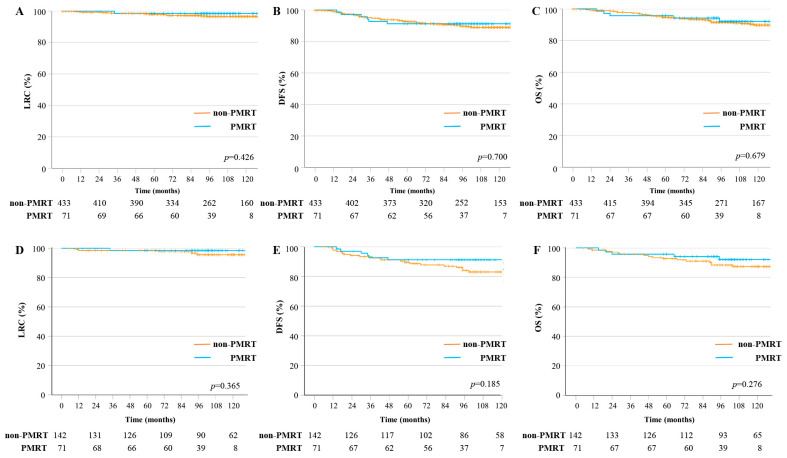
Treatment outcomes according to the use of PMRT: (**A**) LRC, (**B**) DFS, and (**C**) OS rates before PSM, and (**D**) LRC, (**E**) DFS, and (**F**) OS rates after PSM. DFS, disease-free survival; LRC, loco-regional control; OS, overall survival; PMRT, post-mastectomy radiotherapy; PSM, propensity score matching.

The median follow-up period for the whole study population was 105 months (interquartile range: 85.0–129.8). For the PMRT group, the median follow-up period was 99 months, while that of the non-PMRT group was 109 months before PSM and 116.5 months after PSM, respectively. During the follow-up period, ten (2.0%) patients developed chest wall recurrence: one (0.2%) patient in the PMRT group, and nine (1.8%) patients in the non-PMRT group. Regional recurrence was observed in 11 (2.2%) patients: 1 (0.2%) patient in the PMRT group and 10 (2.0%) patients in the non-PMRT group. The IMNs were the most common site of regional recurrence (*n* = 5), followed by the supraclavicular LNs (*n* = 4) and the axillary LNs (*n* = 4). Before PSM, the 8-year LRC, DFS, and OS rates in the non-PMRT and PMRT groups were 98.5% and 96.5% (*p* = 0.426), 89.7% and 91.2% (*p* = 0.700), and 91.5% and 92.1% (*p* = 0.679), respectively (Figure 1A–C). In the PSM cohort (*n* = 213), four (5.6%) patients in the non-PMRT group and one (1.4%) patient in the PMRT group had chest wall and regional recurrence. After PSM, the 8-year LRC, DFS, and OS rates in the non-PMRT and PMRT groups were 95.6% and 96.5% (*p* = 0.365), 84.1% and 91.2% (*p* = 0.185), and 88.4% and 92.1% (*p* = 0.276), respectively (Figure 1D–F).

### 3.3. Treatment Outcomes According to Prognostic Factors

As shown in Table 2, three LN metastases were identified as a significant risk factor for LRC and DFS rates and LVI for OS rate.

**Figure 2:**
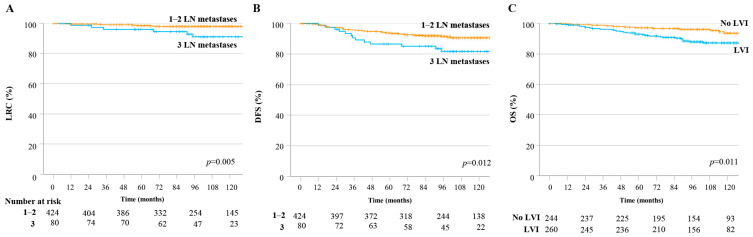
LRC, DFS, and OS rates according to the presence of risk factors in the overall study population. (**A**) LRC rate and (**B**) DFS according to the number of LN metastases. (**C**) OS according to the presence of LVI. DFS, disease-free survival; LN, lymph node; LRC, loco-regional control; LVI, lympho-vascular invasion; OS, overall survival.

Figure 2 shows that treatment outcomes differed significantly according to the prognostic factors identified by the Cox proportional hazards model. The 8-year LRC rate in the 1–2 LN metastasis group was 97.9%, whereas that in the group with three LN metastases was 90.9% (*p* = 0.005; Figure 2A). The 8-year DFS rate was also significantly different between the 1–2 LN metastasis group and the group with three LN metastases (91.5% vs. 81.7%, respectively; *p* = 0.012) (Figure 2B). The 8-year OS rate in patients with LVI was significantly lower than that in patients without LVI (87.8% vs. 96.0%, respectively; *p* = 0.011) (Figure 2C).

### 3.4. Treatment Outcomes According to the Use of PMRT

The treatment outcomes according to the use of PMRT in patients with risk factors were compared in the whole cohort, and PMRT did not significantly improve treatment outcomes regardless of the presence of risk factors. The 8-year LRC rates in patients with three LN metastases were 87.1% (non-PMRT group) vs. 96.6% (PMRT group) (Figure S1A, *p* = 0.233), and the DFS rates were 78.9% (non-PMRT group) vs. 86.2% (PMRT group) (Figure S1B, *p* = 0.500). The 8-year OS rates of patients with LVI were 86.8% (non-PMRT group) vs. 91.1% (PMRT group) (Figure S1C, *p* = 0.332), showing no significant benefit in treatment outcomes after PMRT. To evaluate whether patients without prognostic factors benefitted from PMRT, the treatment outcomes were compared in the low-risk group, as shown in Figure S2. In patients with 1–2 LN metastasis, the 8-year LRC rates were 97.7% (non-PMRT group) vs. 100% (PMRT group) (Figure S2A, *p* = 0.348), and the DFS rates were 91.1% (non-PMRT group) vs. 94.9% (PMRT group) (Figure S2B, *p* = 0.416). The 8-year OS rates in patients without LVI were 96.1% (non-PMRT group) vs. 94.7% (PMRT group) (Figure S2C, *p* = 0.937), which is also not a significant result.

### 3.5. RT-Related Toxicities

Among the 71 patients who received PMRT, 23 patients (32.4%) visited the department of rehabilitation due to arm lymphedema, and 2 patients among them experienced grade 3, which is more than 30% difference in arm circumferences. The incidence rate of lymphedema in the non-PMRT group was 12.0% (52/433 patients), which was lower than the PMRT group with statistical significance (*p* < 0.001). Two patients experienced angina pectoris after 5 and 9 years of PMRT, respectively, and one patient was diagnosed with atrial fibrillation after 7 years of PMRT. There were two patients of subclinical hypothyroidism, and only one patient demonstrated grade 1 radiation pneumonitis. No secondary malignancies related to PMRT were detected during the follow-up period.

## 4. Discussion

We analyzed the treatment outcomes of patients with T1-2N1 breast cancer who underwent total mastectomy. We found no significant difference in 8-year LRC and DFS rates according to the use of PMRT before and after PSM. While there was no definite evidence of benefits in treatment outcomes, the PMRT group had a higher incidence of arm lymphedema than the non-PMRT group. Three LN metastases were identified as a prognostic factor for LRC and DFS rates and LVI for OS rate. However, the benefit of PMRT was not clearly shown even in patients with such a risk factor. Based on these backgrounds, we insist that future prospective studies stratifying the risk group who needs PMRT should be conducted in the context of re-evaluation of the role of PMRT in T1-2N1 breast cancer patients.

Classic evidence supporting the use of PMRT in patients with T1-2N1 breast cancer has demonstrated improved loco-regional control and survival. The EBCTCG meta-analysis demonstrated the value of PMRT in patients treated between 1964 and 1986, reducing the 10-year LRR rate from 20.3% to 3.8% and increasing the 20-year OS rate from 49.8% to 57.7% [2]. Subgroup analysis of the Danish Breast Cancer Group 82 b & c trial demonstrated a reduction in the 15-year LRR rate after PMRT from 27.0% to 4.0% and an improvement in the 15-year OS rate from 48.0% to 57.0% [14]. However, the conclusions have been criticized for the inadequate axillary surgery, which is inconsistent with the current standard. In addition, with the recent development of systemic therapies (taxane-based chemotherapy and HER2-targeted therapy), patients have shown a 10-year loco-regional control rate of >90%, even without PMRT, highlighting the need for the re-evaluation of the role of PMRT along with improving treatment outcomes [15,16,17,18]. Considering that the major clinical trials supporting PORT in N1 breast cancer patients had not reflected the advances of such systemic therapies [5], the idea of de-escalation or individualized application of PMRT should be repeatedly asserted in the modern systemic treatment era.

Several series of non-randomized studies have provided insights into the de-escalation of PMRT, demonstrating favorable treatment outcomes even without PMRT. Approximately 800 patients who met the inclusion criteria of the Z0011 trial were analyzed at the Memorial Sloan Kettering Cancer Center, and the regional recurrence rate was low enough (cumulative 5-year LRR rate was 0.7% in the no-RT group, and nodal recurrence rate was 1% in the RT group) to omit RNI in the T1-2N0 or 1–2 LN metastasis group [19]. Moreover, a subgroup analysis of the Breast International Group 02–98 trial suggested that the use of PMRT in patients with T1-2N1 breast cancer should be individualized, challenging the routine use of adjuvant RT after mastectomy [20]. Therefore, efforts to stratify risk groups of patients to superselect the indication of PMRT in T1-2N1 patients have been made. A prognostic model was proposed in our institution for patients with N1 breast cancer to identify high-risk patients who may benefit from PMRT [21]. Patients with T2-stage disease, LVI, extracapsular extension, tumor grade, and non-luminal subtypes were considered to be at high risk of treatment failure, and patients with two or more risk factors were considered potential candidates for RNI. The Korean Radiation Oncology Group 14–23 trial analyzed >1000 patients of pT1-2N1 breast cancer patients and stratified them according to risk factors [22]. Six potential risk factors of close resection margin, age <35 years, T2 stage, high tumor grade, triple-negative biological subtype, and two or three positive lymph nodes were revealed, and the treatment outcomes according to the subgroups stratified by the number of risk factors were as follows; the 5-year LRR rates were 3.6% with 0–1 (*n* = 606), 7.5% with 2–3 (*n* = 655), and 12.7% with 4–6 (*n* = 93) risk factors. However, these two Korean studies did not confirm improved treatment outcomes after PMRT even in selected high-risk patients. Consistent with the previous literature, the present study also could not find evident benefits of PMRT in patient subgroup with the potential risk factor of three LN metastases for LRC/DFS rates and LVI for OS rates, adding evidence to support the de-escalation of PMRT. The results of the randomized phase III SUPREMO (NCT00966888) trial, which evaluated the role of PMRT in intermediate-risk patients with T1–2N0–1 breast cancer, are expected to be published in 2023.

If PMRT can be omitted without compromising oncological safety, it will improve patients’ quality of life and reduce treatment-related toxicities. A major treatment-related toxicity is arm lymphedema, and tri-modality treatment, including RT, is known to influence the incidence of this toxicity [23]. Axillary reverse mapping has recently demonstrated that the upper lateral level I axilla drains the arm but not the breast, and sparing the lower axillary region from radiation is expected to reduce the risk of lymphedema [24,25]. Our result also demonstrates that adding PMRT could increase the risk of arm lymphedema when compared to the non-PMRT group, which emphasizes the need for individualized application of PMRT to reduce this RT-related toxicity. Proton therapy could be one of the efforts because it could result in improved target control while sparing normal organs, such as the heart and lungs, or non-target axillary regions [26,27]. Consequently, the risk of radiation-related toxicities, such as cardiac toxicity, radiation pneumonitis, or secondary malignancies, which has been remained an important concern in breast cancer patients, could be lowered [28,29,30]. In our study, three cardiac events including angina pectoris and arrhythmia were found after PMRT, even though the correlation between PMRT and these events is not evident. Although neither severe radiation pneumonitis nor secondary malignancies were detected in the present study, treatment de-escalation seems reasonable and worth considering based on this background, when comparable treatment outcomes are guaranteed.

Our study has some limitations. First, although we performed PSM to control for selection bias and confounding patient characteristics arising from the retrospective nature of the study, potential confounding factors may still exist. Second, the small number of study participants and LRR events may limit the reliability of our analyses and reduce the statistical power, potentially leading to type II errors when finding non-significant differences. Therefore, special caution is required when interpreting and applying our results in the clinical setting. We included patients who underwent mastectomy between 2006 and 2014 to ensure a sufficient follow-up period and to include patients who received taxane-based modern systemic therapies. In addition, we excluded patients who received immediate reconstruction after mastectomy, which made it difficult for our study to include a sufficient number of patients. Therefore, future prospective studies to validate our thesis are strongly requested.

## 5. Conclusions

In conclusion, patients with T1–2N1 breast cancer showed favorable LRC rate regardless of the use of PMRT. In addition, the benefit of PMRT was not evident even in selected patients with potential risk factors such as three LN metastases and LVI, adding evidence to support the de-escalation and individualized application of PMRT. We insist that further prospective trials are needed to specify and re-evaluate the indications and the role of PMRT in the era of standard systemic therapy.

## Figures and Tables

**Table 1:** Patient and tumor characteristics before and after PSM.

	Total (*n* = 504)	Before PSM			After PSM		
		Non-PMRT (*n* = 433)	PMRT (*n* = 71)	*p*	Non-PMRT (*n* = 142)	PMRT (*n* = 71)	*p*
Age (years, range)	Median 49.0 (24–84)	Median 48 (32–74)	Median 49 (24–84)		Median 48 (32–74)	Median 48 (31–80)	
Menopause				0.395			0.766
Pre-	289 (57.3)	245 (56.6)	44 (62.0)		85 (59.9)	44 (62.0)	
Post-	215 (42.7)	188 (43.4)	27 (38.0)		57 (40.1)	27 (38.0)	
Laterality				0.841			0.771
Rt.	250 (49.6)	214 (49.4)	36 (50.7)		67 (47.2)	36 (50.7)	
Lt	254 (50.4)	219 (50.6)	35 (49.3)		75 (52.8)	35 (49.3)	
Location				0.448			0.530
Laterally confined	169 (33.5)	146 (33.7)	23 (32.4)		55 (38.7)	23 (32.4)	
Inner/central	139 (27.6)	123 (28.4)	16 (22.5)		34 (23.9)	16 (22.5)	
Multicentric	196 (38.9)	164 (37.9)	32 (45.1)		53 (37.3)	32 (45.1)	
Multifocality				0.042			0.114
No	317 (62.9)	280 (64.7)	37 (52.1)		90 (63.4)	37 (52.1)	
Yes	187 (37.1)	153 (35.3)	34 (47.9)		52 (36.7)	34 (47.9)	
Pathology				0.457			0.124
IDC	456 (90.5)	394 (91.0)	62 (87.3)		134 (94.4)	62 (87.3)	
ILC	21 (4.2)	18 (4.2)	3 (4.2)		4 (2.8)	3 (4.2)	
Others	27 (5.3)	21 (4.8)	6 (8.5)		4 (2.8)	6 (8.5)	
Grade				0.026			0.282
Low	77 (15.3)	73 (16.9)	4 (5.6)		31 (21.8)	4 (5.6)	
Intermediate	262 (52.0)	225 (52.0)	37 (52.1)		62 (43.7)	37 (52.1)	
High	165 (32.7)	135 (31.2)	30 (42.3)		49 (34.5)	30 (42.3)	
LVI				<0.001			0.831
Negative	244 (48.4)	224 (51.7)	20 (28.2)		42 (29.6)	20 (28.2)	
Positive	260 (51.6)	209 (48.3)	51 (71.8)		100 (70.4)	51 (71.8)	
Ki-67				0.858			0.43
1+/2+	406 (80.6)	350 (80.8)	56 (78.9)		105 (73.9)	56 (78.9)	
3+/4+	98 (19.4)	83 (19.2)	15 (21.1)		37 (26.1)	15 (21.1)	
ER				0.152			0.452
Negative	111 (22.0)	100 (23.1)	11 (15.5)		28 (19.7)	11 (15.5)	
Positive	393 (78.0)	333 (76.9)	60 (84.5)		114 (80.3)	60 (84.5)	
HER2 status				0.82			0.586
Negative	360 (71.4)	310 (71.6)	50 (70.4)		105 (73.9)	50 (70.4)	
Positive	142 (28.2)	121 (27.9)	21 (29.6)		37 (26.1)	21 (29.6)	
Unknown	2 (0.4)	2 (0.5)	0 (0.0)		0	0 (0.0)	
Subtype				0.463			0.492
HR+	391 (77.6)	331 (76.4)	60 (84.5)		114 (80.3)	60 (84.5)	
HER2+	64 (12.7)	57 (13.2)	7 (9.9)		13 (9.2)	7 (9.9)	
TNBC	47 (9.3)	43 (9.9)	4 (5.6)		15 (10.6)	4 (5.6)	
Unclassified	2 (0.4)	2 (4.6)	0 (0.0)		0 (0.0)	0 (0.0)	
T stage				0.009			0.911
T1	199 (39.5)	181 (41.8)	18 (25.4)		35 (24.6)	18 (25.4)	
T2	305 (60.5)	252 (58.2)	53 (74.6)		107 (75.4)	53 (74.6)	
Number of dissected LNs	19 (1~44)	18 (1~43)	21 (3~44)	0.063	20 (9~37)	21 (3~44)	0.622
Number of LN metastases				<0.001			0.282
1	288 (57.1)	276 (63.7)	12 (16.9)		31 (21.8)	12 (16.9)	
2	136 (27.0)	107 (24.7)	29 (40.8)		62 (43.7)	29 (40.8)	
3	80 (15.9)	50 (11.5)	30 (42.3)		49 (34.5)	30 (42.3)	
RM				0.784			0.426
Negative	445 (88.3)	383 (88.5)	62 (87.3)		129 (90.8)	62 (87.3)	
Close	59 (11.7)	50 (11.5)	9 (12.7)		13 (9.2)	9 (12.7)	
Axillary management				0.031			0.497
SLNB	79 (15.7)	74 (17.1)	5 (7.0)		14 (9.9)	5 (7.0)	
ALND	425 (84.3)	359 (82.9)	66 (93.0)		128 (90.1)	66 (93.0)	
Adjuvant therapy							
Anthracycline	426 (84.5)	364 (84.1)	62 (87.3)	0.482	129 (90.8)	62 (87.3)	0.426
Taxane	432 (85.7)	364 (84.1)	68 (95.8)	0.009	136 (95.8)	68 (95.8)	1
Anti-HER2	127 (25.2)	107 (24.7)	20 (28.2)	0.607	37 (26.1)	20 (28.2)	0.743
Endocrine	393 (78.0)	332 (76.7)	61 (85.9)	0.082	117 (82.4)	61 (85.9)	0.513

PSM, propensity score matching; ALND, axillary LN dissection; ER, estrogen receptor; HER2, human epidermal growth factor receptor 2; HR, hormone receptor; IDC, invasive ductal carcinoma; ILC, invasive lobular carcinoma; LN, lymph node; LVI, lympho-vascular invasion; PMRT, post-mastectomy radiotherapy.

**Table 2:** The Cox proportional hazards model for LRC rate, DFS, and OS in the overall study population.

Variables	LRRFS				DFS				OS			
	UVA	MVA			UVA	MVA			UVA	MVA		
	*p*	*p*	HR	95% CI	*p*	*p*	HR	95% CI	*p*	*p*	HR	95% CI
Age (yrs)	0.771				0.274				<0.001			
Age (≤50 vs. >50)	0.5				0.308				0.014	0.716	1.198	0.453–3.172
Menstruation (Pre vs. Post)	0.466				0.374				0.004	0.162	2.008	0.756–5.336
Location (Medial/multicentric vs. Laterally confined)	0.075	0.056	0.137	0.018–1.050	0.366				0.614			
Multifocality (No vs. Yes)	0.213				0.268				0.049	0.103	0.553	0.271–1.127
Grade (Low/Int vs. High)	0.529				0.123				0.148			
Subtype (HR+ vs. HER2 +)	0.399				0.053	0.112	0.298	0.105–1.124	0.983			
Subtype (HR+ vs. TNBC)	0.162				0.963				0.108			
pT stage (1 vs. 2)	0.159				0.118				0.026	0.076	1.869	0.936–3.732
Number of LN metastasis (1, 2 vs. 3)	0.010	0.005	4.619	1.6–13.338	0.014	0.02	2.1	1.126–3.917	0.099	0.261	1.489	0.743–2.982
Extranodal extension (No vs. Yes)	0.897				0.138				0.574			
LVI (No vs. Yes)	0.333				0.098	0.132	1.555	0.875–2.761	0.013	0.023	2.127	1.112–4.070
Resection margin (Negative vs. Close)	0.267				0.334				0.911			

LRC, loco-regional control; DFS, disease-free survival, OS overall survival; UVA, univariate analysis; MVA, multivariate analysis; HR, hazard ratio; CI, confidence interval; HR, hormone receptor; HER2, human epidermal growth factor receptor 2; TNBC, triple-negative breast cancer; LN, lymph node; LVI, lympho-vascular invasion.

## Data Availability

Research data are stored in an institutional repository and will be shared upon request to the corresponding author.

## Supplementary Materials

The following supporting information can be downloaded at https://www.mdpi.com/article/10.3390/cancers15225473/s1. Figure S1: LRC, DFS, and OS rates in subgroup with prognostic factors according to the application of PMRT before PSM; Figure S2: LRC, DFS, and OS rates in subgroup without prognostic factors according to the application of PMRT before PSM
