# Supplementary figures and images for: The Role of Post-Mastectomy Radiotherapy in T1-2N1 Breast Cancer Patients: Propensity Score Matched Analysis
Source: Cancers (Basel). 2023 Nov 19;15(22):5473. doi: 10.3390/cancers15225473 (PMC10670498; doi:10.3390/cancers15225473)

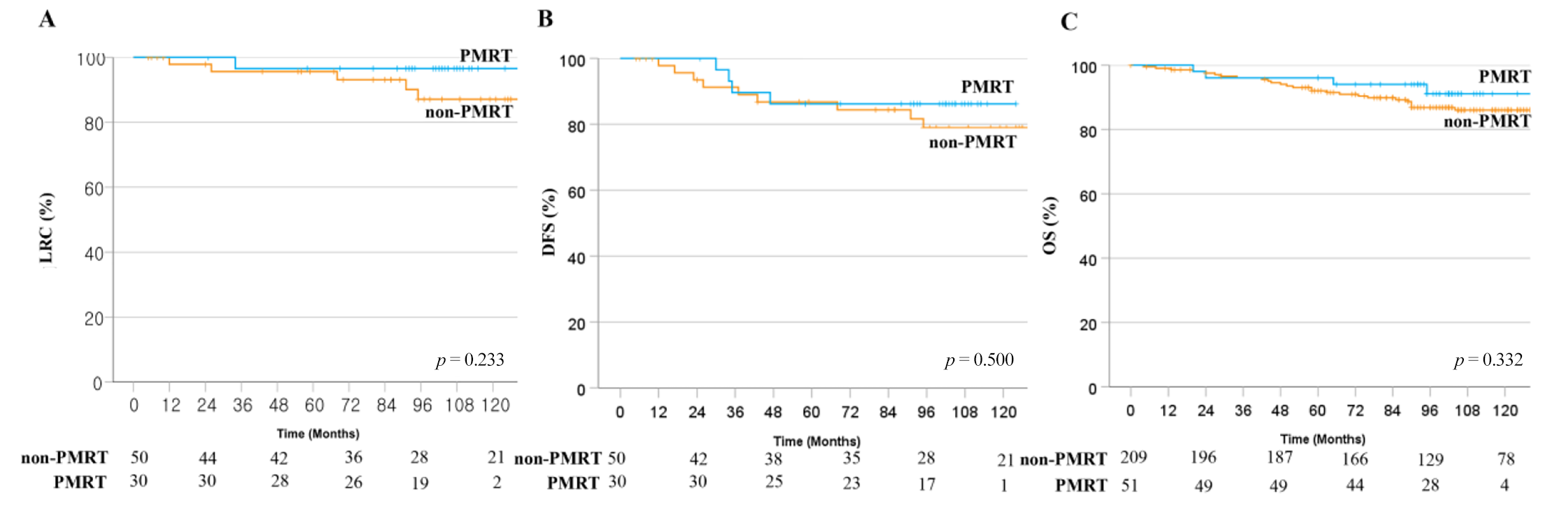

Supplement: Supplementary file 1 [file cancers-15-05473-s001.zip › Supple Figure S1.tif]

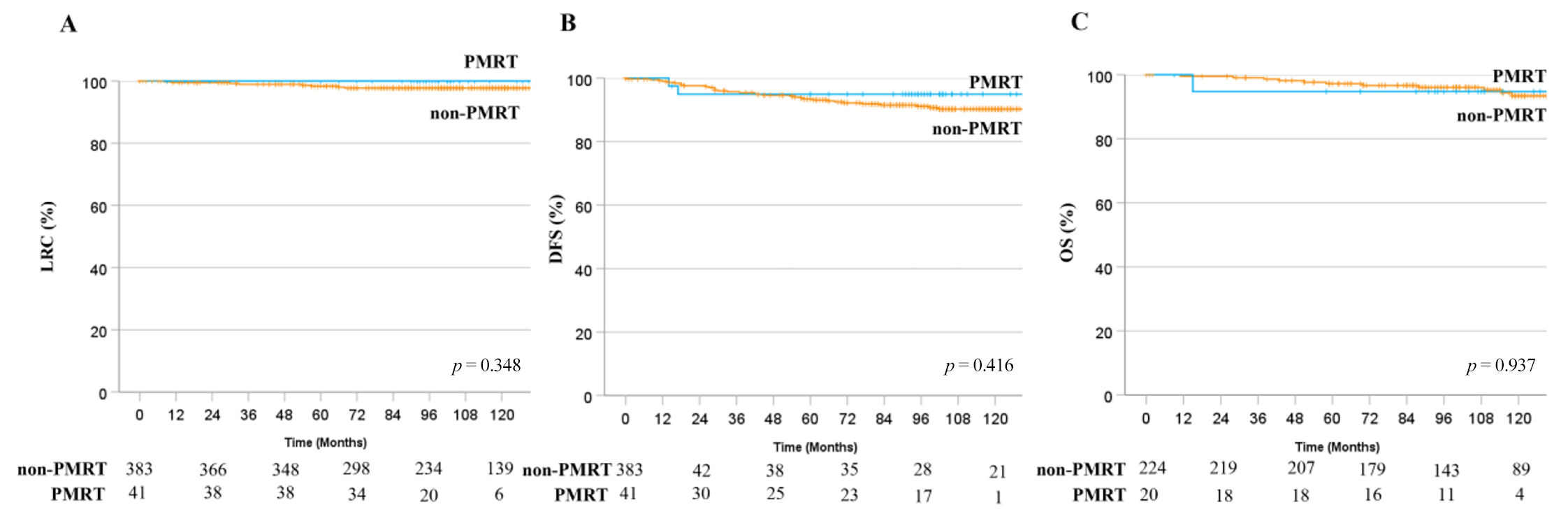

Supplement: Supplementary file 1 [file cancers-15-05473-s001.zip › Supple Figure S2.tif]
